# Experiences and Perspectives of the COVID-19 Pandemic and Vaccinations in Culturally and Linguistically Diverse (CALD) Populations in Australia: A Qualitative Study

**DOI:** 10.7759/cureus.74463

**Published:** 2024-11-25

**Authors:** Tasnia Rafi, Mohammad A Rahman

**Affiliations:** 1 Public Health, Oceania University of Medicine, Melbourne, AUS; 2 General Practice, RediCare Medical Centre, Melbourne, AUS

**Keywords:** covid-19, covid-19 vaccination hesitancy, culturally and linguistically diverse, health inequalities, public health policy

## Abstract

Qualitative research surrounding the impacts of COVID-19 and vaccine hesitancy has been extensively studied in the European context; however, limited research has been conducted within communities in the Australian context. This research paper highlights the issues experienced by culturally and linguistically diverse (CALD) members during the COVID-19 pandemic and vaccination rollout. The purpose of this study is to strengthen our understanding of the challenges experienced by CALD communities and enable healthcare policies to be developed and implemented to prevent these communities from being disadvantaged in a healthcare crisis. The methods of this study include focus group sessions with 12 participants from CALD backgrounds across different states including Victoria, Northern Territory, and Queensland. They were divided into two separate Zoom sessions and aimed to amplify the voices in Victoria, as the state had the nation's longest and most challenging COVID-19 lockdown laws. Key findings of the study highlighted the language barriers, racism, and lack of cultural awareness experienced among CALD communities during the pandemic. Additionally, there was a significant division in views and experiences within CALD communities and families. Social media played a prominent role in dividing the perceptions and understanding of health information during the pandemic. Participants were motivated to be vaccinated due to workplace protocols or to protect their family members and move toward normality. Lastly, the government's mandatory vaccination policies limited personal choice, resulting in a loss of skilled workers and pressured some participants into making healthcare decisions within a limited period of time. The study's findings reflect that the CALD communities and families were disproportionately impacted during the COVID-19 pandemic and vaccination rollout, further highlighting and adding to the health inequities among CALD communities in Australia.

## Introduction

Qualitative research surrounding vaccine hesitancy and the impacts of COVID-19 among the refugee and migrant communities has been extensively studied over the last two years. However, these studies are predominantly in the setting of the European context, notably the United Kingdom (UK) [1-4].

Experiences of young people from migrant or refugee backgrounds have been documented independently on a smaller scale through not-for-profit organizations, such as the Multicultural Youth Advocacy Network (MYAN) [5]. Additionally, in 2020, before the nationwide lockdown, a national cross-sectional online study was conducted among 1,420 Australian adults [6]. General perceptions toward the COVID-19 vaccination rollout programs were positive, with 83% agreeing that the vaccines would be effective and valuable for disease prevention; 84% of the participants also agreed that it was beneficial to follow government guidelines regarding COVID-19 vaccination [6]. However, a lack of culturally and linguistically diverse (CALD) voices was notable at the time, and qualitative studies have slowly been emerging over the last two years, particularly among the refugee and migrant populations in Australia [7-9]. Some key findings reported in these studies included the challenges around health or vaccine literacy, the availability of trustworthy resources, and logistical barriers to accessing the healthcare system, which impacted the understanding and decisions around the COVID-19 virus and vaccination policies. However, there are limited studies focusing on participants from Victoria, particularly metropolitan Melbourne, which had the most extended lockdown period compared to other states and on a global scale.

Vaccine hesitancy is not a new phenomenon among CALD communities worldwide. Evidence suggests that many refugees and migrants have been hesitant about measles, influenza, or human papillomavirus (HPV) vaccinations in the past [1,10]. Over time, it has become evident that cultural or religious views are not the only reasons for vaccine hesitancy [10]. Discrimination and marginalization have also been the root of many health implications among the refugee and migrant community, which is no different in the setting of a worldwide pandemic [2,10]. Unfortunately, the mistreatment of these communities by the government and healthcare systems has played a significant role in the lack of uptake of COVID-19 vaccinations [1,2]. For example, in the UK, the treatment toward migrants, particularly undocumented migrants, had worsened when patient data sharing agreements between the Home Office of Immigration and Health Services were implemented, which made many migrants fearful and distrustful toward preventative health services during the pandemic due to the fear of deportation [2].

Furthermore, the digitalization of healthcare and inconsistent changes to new health information during a pandemic have led to additional challenges in many CALD communities [4]. Issues around the lack of culturally appropriate digitalized healthcare, confidentiality, and technology literacy may have further exacerbated the ability to seek appropriate healthcare during a pandemic, according to studies across the UK [2,4].

This study aims to understand the experiences and challenges of adults aged 18-35 of CALD backgrounds during the COVID-19 pandemic in Australia. Given the varying impact of the COVID-19 pandemic across different states in Australia, there is a need for analysis to understand its effects on diverse communities nationwide. The study also aimed to include participants from Melbourne, Victoria, as this state experienced the longest lockdown in Australia and around the world, with a total of 267 days from March 2020 to October 2021 [11], unlike other larger states such as New South Wales (NSW), which had a total of 106 days spent in lockdown [12]. While the general population of Australia has encountered challenges during the pandemic, it is crucial to recognize the additional hurdles faced by CALD communities that have historically been disadvantaged in urgent healthcare settings. The findings of this study will assist public health policymakers in incorporating policies that are effective and culturally appropriate for CALD members of our community.

## Materials and methods

Participants

Study Design

The study design is a qualitative study using interactive focus group sessions through Zoom with discussions about the experiences of the COVID-19 pandemic and vaccination rollout across Australia.

Sampling Technique

The study used a snowball sampling method starting from members who were previously affiliated with community organizations, including the Multicultural Youth Advocacy Network (MYAN) or Centre for Multicultural Youth (CMY), as well as public university forums and discussion applications. With this method of sampling, an initial participation rate of 35% was reached, and eventually, an overall participation rate of 52% was achieved over the recruitment period from the total number of individuals approached or invited. The aim was to reach at least 12 participants as a sample size between six and 12 has been theorized to be the best for reaching data saturation in a focus group setting [13].

Inclusion Criteria

The inclusion criteria included people aged 18-35 who were either skilled migrants, refugees, asylum seekers, undocumented, or diaspora of migrant and refugee families across Australia. This age demographic reflects young adults with higher social mobility, which can increase their exposure risk to healthcare policies given the different stages of education, work, social commitments, and travel. Additionally, this age group has a higher connectivity to the digital world of social media, and the ability to reflect on these experiences is more prevalent among this age group. Participants were required to have lived in Australia for at least two years before 2022, aligning with the timeline of the COVID-19 pandemic.

Final Sample and Recruitment Period

The recruitment period lasted approximately three months, and a sample size of 12 (eight female participants and four male participants) was yielded. Participants were from Victoria, Queensland, and the Northern Territory. The participant demographic is reported in Table 1.

**Table 1:** Demographic characteristics of participants

Participant number	Gender	Migrant status	Previous or current healthcare worker	State and city of residence during 2020-2022
1	Female	Czech Republic/South African diaspora of skilled migrant and refugee families	Yes	Brisbane, Queensland
2	Female	North Indian diaspora of skilled migrant families	Yes	Melbourne, Victoria
3	Female	Skilled migrant of Indian background	Yes	Melbourne, Victoria
4	Male	Czech Republic/South African diaspora of skilled migrant and refugee families	No	Brisbane, Queensland
5	Male	South African/New Zealand diaspora of skilled migrant families	No	Brisbane, Queensland
6	Female	Skilled migrant of Kuwaiti/Palestinian background	Yes	Melbourne, Victoria
7	Female	Bangladeshi diaspora of skilled migrant families	Yes	Melbourne, Victoria
8	Male	Skilled migrant of Malaysian Chinese background	Yes	Melbourne, Victoria
9	Female	Refugee from Africa	Yes	Darwin, Northern Territory
10	Female	Skilled migrant of Malaysian Chinese background	No	Melbourne, Victoria
11	Female	Bangladeshi diaspora of skilled migrant families	No	Melbourne, Victoria
12	Male	Skilled migrant of Malaysian Chinese background	Yes	Melbourne, Victoria

Procedure

Ethics approval was obtained through the Ethics Research Committee of the Oceania University of Medicine with Institutional Review Board (IRB) number 22-0308TR. Focus group discussions were conducted by one interviewer (the lead author) over a Zoom session, as participants were from interstate. All focus group sessions were conducted in English, and no interpreter was used. The focus group sessions were divided into two groups to facilitate the availability of participants and for more accessible data collection and management. Each focus group session was conducted for 60-90 minutes. Consent forms were obtained from all participants of the session. All Zoom sessions were recorded, and participants' transcripts remained anonymous and were used for qualitative analysis. Previous literature was used to guide important themes to cover in the interview questions, including the "Three C's" model [2,10]. The Three C's include convenience (access to health information and transport), complacency (belief around the viruses and severity), and confidence (in the health system and government) [2,10]. The questions were tailored to the participants' different state policies using this model, and additional questions about personal experiences from both a community and personal perspective during the COVID-19 lockdowns and vaccination rollout were developed. The focus group questions were a combination of open-ended and follow-up closed-ended questions. A research advisor reviewed the interview questions to confirm that the questions aligned with the intended themes and provided further refinement of questions as required. The interview schedule began with an introductory section for the moderator to explain the session and for participants to introduce themselves and build rapport between the moderator and the participants. The schedule was flexible, and probing questions were also used to engage in further discussion and include the input of all participants in the study. Reimbursement measures included 49 dollars for each participant's input for the study.

Analysis

The study used a grounded theory approach, as it aimed to develop theories from the focus group data rather than conducting the study with an initial hypothesis. Continuous data collection and analysis in an iterative manner until theoretical saturation is reached allows theoretical insights to develop from the data collected [14]. The authors read through the transcript data, which were cross-checked by another investigator (a research advisor) for reading and reflection to review any bias and refine emerging concepts found in the data. Once the transcripts were read, cross-checked, and cleaned, they were coded using NVIVO 14 software. The data was coded using Adu's qualitative data coding guide [15], starting with open coding to highlight a series of thematic codes, which were further analyzed into core categories with axial coding. This way of coding was used to generate central themes and connect appropriate codes accordingly. The research advisor reviewed the coding process and themes generated to maintain credibility.

## Results

Themes

**Figure 1:**
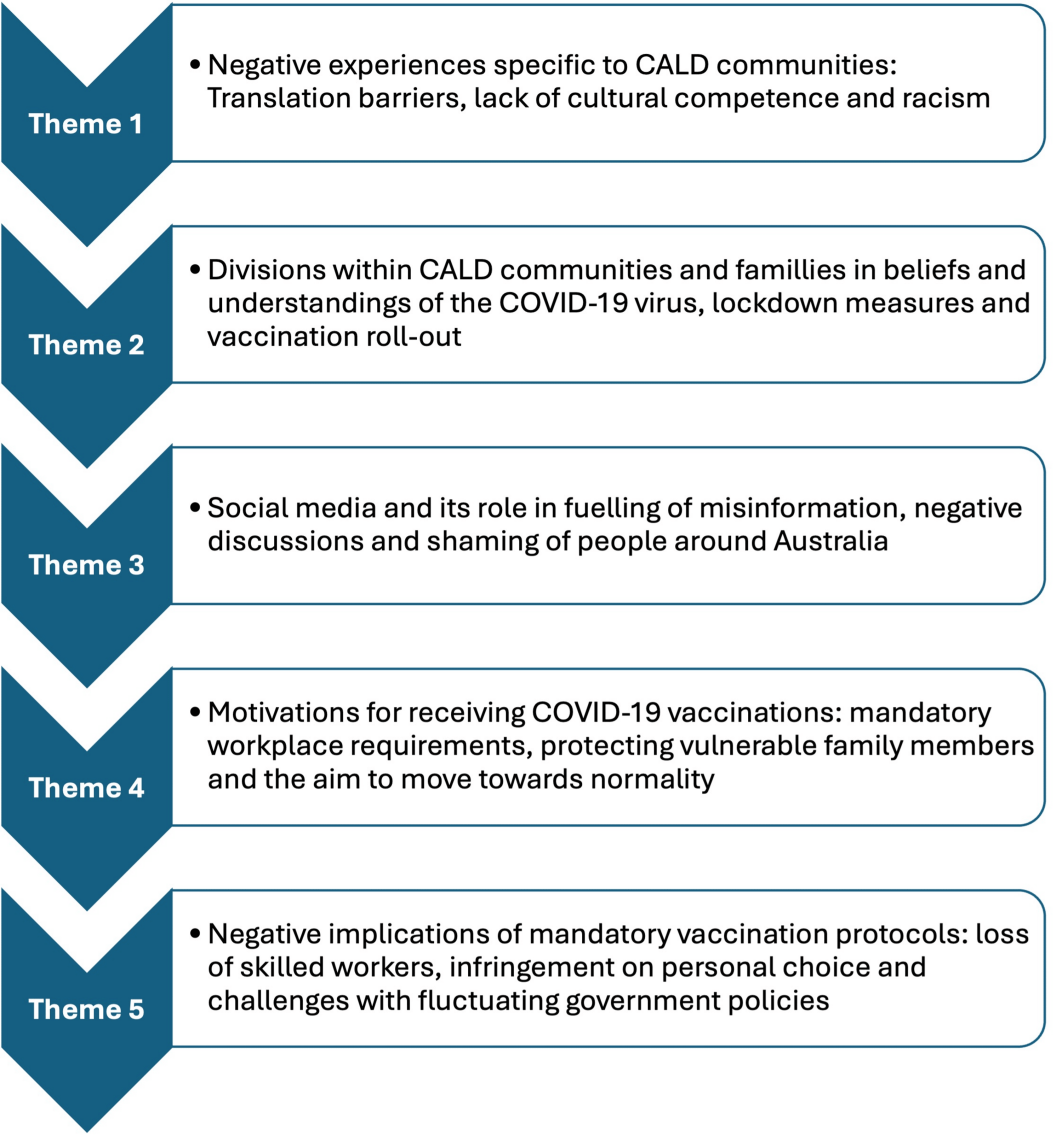
Main themes identified from the COVID-19 pandemic and vaccination rollout experiences COVID-19: coronavirus disease 2019, CALD: culturally and linguistically diverse

The study concluded with five central themes highlighted in Figure 1.

Negative experiences specific to CALD communities: translation barriers, lack of cultural competence, and racism

Translation Barriers

A common theme of translation barriers was referenced 15 times across the two focus group discussions. The participants discussed that they experienced translation barriers at a personal level, with their family members, and in the context of their work experiences for those who worked in healthcare. One participant highlighted the challenges in managing care for her mother during the pandemic and the strict avoidance of close contact. While the health system itself was not complicated for her and her family to access, the language and cultural barriers were challenging to navigate during her mother's hospital stay, further adding to the stress and lack of cultural nuance in patient care ("… so it was, and she's (mother of participant) also a second language speaker … So she doesn't speak English as a first language … and not having us to be there and explain things on her behalf or with the practitioners was a nightmare" (P6.1)).

Another discussion around translation barriers included bilingual or multilingual participants who noticed the inappropriate translations of their language(s) about the COVID-19 lockdown measures and the information about the COVID-19 vaccination rollout, particularly from the Department of Health materials. The participants reflected that the level of urgency in a pandemic regarding the conveying of appropriate messaging around COVID-19 and the implications of vaccination was not appropriate from their understanding, which could have played a major role in the need to abide by state rules and vaccination hesitancy during the rollout ("… I speak Bengali. So (I) looked at the Bengali translated information coming out of the Department of Health, and all the vowels and the letterings were very different to that to the extent where you could not actually read it. Now, this is coming from our Department of Health; you would expect them to have the resources and the urgency to have a proper translation formed in the right way so that people in that community could read it" (P7.1)).

Lack of Cultural Awareness and Challenges With Limited Health Literacy

Furthermore, several participants highlighted that having CALD workers in the medical workforce or healthcare workers with better cultural competence would have alleviated a lot of the challenges in managing patients who come from diverse cultural backgrounds and could have provided better education and information to communities that were not able to comprehend the issues in English ("… a lot of the times, because medical practitioners do not necessarily have the cultural guidance or the cultural practice. So, there is this distrust that forged medical fears and multicultural communities and … I definitely felt there was a bit of a pushback from a lot of communities" (P7.2)).

Participants who spoke English and worked in healthcare felt that they had the privilege of health and English literacy, which unfortunately did not apply to members of their families or communities in many settings during the pandemic ("… I think because of the health literacy, the health background that I come from, it definitely made things a lot easier for me in choice, but I could definitely see where he (participant's father) was coming from with his thinking" (P1.1)).

Racism and Disadvantage to CALD Communities

Furthermore, many participants reported that they felt that CALD communities were disadvantaged in many ways throughout the COVID-19 pandemic. One participant highlighted the negative racist connotations that the pandemic had brought out to the broader community, where racist ideologies were used to weaponize Asian communities ("I think around that time, there was a lot of this racist idea going around … like, a Chinese virus … which was quite … difficult to see as well, because … being from a minority community … being an immigrant … seeing how it was impacting the minorities in Australia was quite shocking" (P2.1)).

The Victorian participants also reflected on their views on the coerced government building lockdown in 2020. There were concerns about the misuse of power by the Victorian government by targeting people in government housing, particularly those from refugee and migrant backgrounds living in high-density areas. While the justification of lockdowns for high-density areas was reported to be valid, the lack of communication and short notice for communities with already limited resources reflected the unfairness of the lockdown measures for minorities compared to other areas across metropolitan Melbourne ("… there was sort of a racial and socio-economic profiling in a way, and therefore … was definitely quite inflammatory that they did that. Understanding the … high density living in those situations, it was that sudden sort of … can't go out that really impacted people. So … if this issue could (have been) communicated and come from the residents within the building, it would have been more acceptable" (P7.3); "… because I was working in the hospital at the time, and what I did was visit patient's homes. And when that happened, we couldn't really visit any patients that were in those community housing. What I felt towards that was I felt like they were disadvantaged from that perspective. They've …got their needs as well. And it's not being met just because they're imposing this rule on them" (P12.1)).

Two out of the 12 participants reported their first-hand experiences with newly arrived refugees in their state. They highlighted the negative implications of the lockdown measures and care for these newly arrived migrants, further highlighting the mental health concerns and unfairness experienced by these people due to the lack of language accessibility and inability to communicate the new healthcare rules that have been in place for newly arrived refugees ("… from a first-hand account, I spoke to a refugee who had just arrived from Sudan … and had to isolate for 14 days because anyone who came in on an international flight … had to isolate for 14 days. And … he did tell me that he was having suicidal thoughts because he was locked in that room for 14 days, with no contact at all with any of his family members or anyone that even knew his language remotely. I also only managed to speak to him (through) an interpreter … I also felt really helpless about the situation because I was talking to him through the phone, and I was trying to offer him as much support as I could. But I guess that wasn't enough" (P3.1)).

Division within CALD communities and families during the lockdown periods and vaccination rollout

A number of participants highlighted divisions between families and their communities regarding the COVID-19 pandemic measures and the need for the COVID-19 vaccination. One reason for the division is personal beliefs and experiences around the freedom of choice for vaccinations by CALD family members ("… so my father was a refugee and left his country of origin (the) Czech Republic, due to (the) Communist regime, and they were not communist. So, they had to flee at that time. If you were not a communist, then they made life very difficult for you. But when, for my family, when we heard that, you know, you have to get the vaccine. My father felt that this was, you know, is this another communist type of thinking, pushing everything on everyone?" (P4.1)).

Mistrust with the circulating health information was referenced eight times across the focus groups when discussing vaccine hesitancy or reluctance among the participants' families and community members. Mistrust was also fueled by the circulation of information from social media as a means of replacing legitimate sources of information for CALD communities because opinions or articles were easily posted by any member of the community. One participant highlighted how misinformation fueled many of their families' disagreements, particularly when conspiracy theories were heightened on social media and in communication streams between different community members ("I think a lot of disagreements were there within my family. Regarding the vaccination … I had a member of my family actually think that the vaccines were microchips that were being inserted by the government just to keep a tab on us … and it was really hard trying to explain to him that this was not the case" (P3.2)).

Two participants reported from their experiences that they felt multicultural communities were deprived of regular interactions and communications. The participants highlighted that CALD communities thrive on discussions and gatherings and often rely on one another for important information due to different languages spoken in the home, which was removed in the setting of the pandemic and further caused negative implications during the COVID-19 pandemic ("… it's like very known that our community … thrives off interactions with each other face to face communications. And just overall life, just having a party … there's the entire family (that) comes together. And it's a very large part of our traditions, and what we what we're about … overall, the communications amongst communities could have been better, especially amongst refugee communities … there's nothing like talking to someone who speaks your native tongue. Like my father, he speaks with his parents because they can barely speak English. And I guess that kind of separation … had an effect on their relationship because they were constantly apart and talking over the phone was nowhere near the same because they're both in their 90s. And they can't really use the phone. So my father really struggled to communicate with his family. And that was really the most detrimental part" (P4.2)).

Social media and its role in fueling misinformation, negative discussions, and shaming of people around Australia

Participants highlighted that social media played a prominent role in understanding the COVID-19 virus, the state government policies around close contacts and isolation protocols, and information regarding the vaccination rollout. Instagram was the most common social media platform used by the participants, and it was referenced 10 times across the two focus groups. However, other platforms, including Facebook, TikTok, Twitter, and the Department of Health, were also reportedly used by participants.

Given the circumstances of the pandemic and lockdown measures, many participants highlighted that social media use became more frequent and often the most common news source for people. Participants reported that social media created confusion regarding the transmission of COVID-19 and the negative impacts of the COVID-19 vaccination. Overall, misinformation from different social media outlets was referenced 19 times across the two focus group discussions in the form of personal opinions, facts from unchecked sources, conspiracy theories, and a tool for shaming members of the community, further fueling confusion around the appropriate health measures of the pandemic ("… it was very much like social media like Instagram, Facebook, as well as like … the news. I think for me, personally, there was a lot of like misinformation, a lot of … opinions on … what's going on with COVID. So, it was really hard to figure out what was actually accurate and what wasn't, especially when the vaccinations came around …" (P2.2); "I think Instagram was a big platform in the sense of how it shaped the perception of the pandemic … it was interesting to see, I guess, I think there was a lot of … shaming … I guess public shaming is probably the best way I would describe it, you know, people getting on there … people being very, very open and … out there with their opinions on you know, go get "vaxxed" (vaccinated) … stay within your five kilometres … all kind of like that kind of shaming and take account type of thing. And … you know, if someone saw (that) their friend … had gone against any of the lockdown rules, then they wouldn't be happy about it. They kind of shamed people for it" (P10.1)).

However, another participant also reflected that community discussions were an outlet for people to discuss their experiences, especially when there was limited evidence supporting the efficacy of the COVID-19 vaccination ("I think COVID … brought a lot of good and bad things, in the sense that there were lots of community discussions going on, on Facebook … people were sharing their experiences and what they're doing. And a lot of young people actually became a bit creative. But the bad things were … not knowing what information they're sharing, you know, some information where it could have been … false. Everyone was just kind of guessing" (P9.1)).

However, most participants agreed that information on social media, through different outlets and personal experiences, made it more challenging to follow lockdown protocols and more difficult to trust the information being relayed. The information continuously changed regarding isolation periods, the number of crowds, mask rules, and discrepancies across state borders, making it more challenging for those who needed to travel interstate ("There (are) only stories about how the government handled things that weren't great. Which also doesn't help with … or trust with what the government is announcing. On top of that, they constantly change the rules, lockdown rules. How many people can be indoor? How many people can be outdoors? Can you wear masks inside or not? Things like that kept changing. So, it became a very confusing, I guess experience, because you don't know which source to trust. Social media, you understand that some of it is just someone's story that can be exaggerated. But at the same time, some of the things that the government puts out from official channels, you don't really know whether you can fully trust as well if they keep going back and forth with their advice" (P12.2); "… when the lockdown was kind of ending, the information was kind of different. Here, they would say, oh, you have to isolate for ten days, or here, they would say you have to isolate for seven days. So, it was kind of not matching, but people used different kinds of platforms to get information about COVID" (P9.2)).

Motivation for receiving COVID-19 vaccinations: mandatory vaccination for employment, protection of vulnerable family members, and the aim to move toward normality

Several participants working in the healthcare sector reported being vaccinated against COVID-19 because their work required mandatory vaccination ("Well, I had to if I wanted to stay employed. So that was, you know, a big reason for me" (P1.2); "… in terms of my personal experience working in healthcare, we did have to have the vaccine by a certain date to work" (P8.1)).

Two other reasons motivated some of the participants to receive the COVID-19 vaccination. The first was their perception of protecting their vulnerable family members at home; they worried they would get their parents or grandparents sick if they did not vaccinate. The second reason for participants accepting vaccination protocols was due to their aim of moving toward normality, particularly given the challenges they faced with their mental and social well-being during the lockdown periods ("… at the time I was living at home with my parents and my parents had a lot of these, I guess, underlying health conditions that they were talking about. And so, for me, it was really important that I didn't put them in any sort of harm or put them in jeopardy if for any reason I got, like, got COVID, I didn't want to pass it on or anything like that. So, for me, it was like for from a protection perspective for myself and (also) for my parents" (P11.1); "yeah, for me, the thing swaying was just that … once we got vaccinated, then … bars … were opening up again … you could go back out to eat … all that kind of stuff … see people. Luckily … no one around me was immunocompromised or anything like that … definitely (I) was just to try and get back to quote unquote … normal life" (P10.2)).

Negative implications of mandatory vaccinations: loss of skilled workers, infringement of personal choices, and challenges with the fluctuation in government policies surrounding COVID-19 vaccination protocols

Participants who worked in the healthcare sector, particularly two participants, felt that the mandatory vaccination for work had led to a loss of skilled workers in the field of healthcare due to their choice of not wanting the mandatory vaccine ("But we did lose a lot of good, educated people, you know, who weren't able to work because of their choices. So even, you know, maybe putting them into other roles, if possible, where they wouldn't have to have that contact with people … I think when someone's been educated for so long, and it's a personal choice … perhaps just to maintain that education and allow it to help the people around us … (and) finding other roles for them" (P1.3)).

Out of all the participants who worked in the healthcare sector, one participant said that they chose not to get vaccinated, given their experiences at the time and knowing people who had had negative implications of the vaccination. Additionally, this participant and other participants felt that there was a particular aspect of coercion, hesitancy, and a lack of information regarding the vaccination status at the time ("I'm not against vaccinations; I had all my vaccinations. But I think what kind of made me not get it was, at that time, I was, you know, heavily pregnant with my daughter. And, and, you know, and there wasn't really clear research into …vaccinations and … pregnancy. So, that was one of the reasons. And on top of that … having close friends who got the vaccination and didn't make it was kind of questionable as well to me … and, aside from that, the fact that they really mandated it … I kind of felt like my power or … my health rights, because … the health rights states that you have the right to refuse … and I kind of felt that our rights were taken away in that sense, that we can't choose to have it or not to have it" (P9.3)).

Additionally, other participants who were vaccinated for work felt that it would have been beneficial if there had been time to make an appropriate decision based on their own research or enough time to be aware of the vaccination's implications, side effects, and efficacy ("… now that I'm thinking about (it), I'm like, oh, you know what, it would have been nice to actually have had some time to think about it and be like … did I actually want it? Because I know like there was anxiety around it as well and a lot of misinformation, as we've already discussed" (P2.3)).

However, most participants agreed that given the uncertainty and difficulty of being in a pandemic for the first time, it could be challenging to determine what could have been done better with the limited resources and understanding of COVID-19 and the vaccine's efficacy ("I'm not sure … what else we could have done. Like I understand, obviously, all the stuff that we're talking about just in terms of translations, and all that all … of course, there (are) things we could have improved, but at the same time … I guess the government was … doing the best that it could, given where we were at, and going through it all together" (P11.2)).

## Discussion

The experiences of poor pandemic planning, fluctuation in state policies, lack of cultural and language awareness, lack of information, and the spread of misinformation throughout the pandemic led participants to highlight why the CALD communities would be impacted negatively compared to the general population. Ultimately, all the themes highlight significant disadvantages within the CALD community during the COVID-19 pandemic. Specifically, the subthemes related to negative experiences of CALD communities (theme 1: translation barriers, cultural awareness, and racism) likely amplified the intensity of the remaining themes related to vaccine hesitancy, social media use, and the decision and outcomes of vaccination within the CALD community. The study further highlights how mistrust exists within CALD families and communities, notably due to the lack of communication and inability to appropriately translate information for a significant portion of the population of Australia. Mandatory vaccination protocols and the loss of skilled workers throughout the pandemic were some of the other identified themes. However, these themes may or may not apply to the general population, as healthcare workers in the study primarily highlighted these issues.

Although not specifically identified as a theme, distrusting the government during the COVID-19 pandemic could be an outcome of the other identified themes. This perspective may have been underexplored, likely due to the selection bias of the study's participant group, and further analysis will be required to address this limitation. However, over the last few years since the COVID-19 lockdowns and vaccination rollout, similar studies in the Australian context have also highlighted that trust in authorities and additionally fear of control have been the main reasons for the hesitancy in receiving the vaccination among CALD communities [7,8].

Social media was an essential aspect of this study as it significantly impacted the beliefs and understanding of many participants. In some cases, it was the only news source they chose to consume, along with their family members, during the COVID-19 pandemic. Given the level of digitalization, access to smartphones, and freedom of speech, news and opinions circulate and can create a grey area in understanding COVID-19 and vaccination efficacy. Additionally, CALD communities were further disadvantaged by needing more appropriate tools through social media to understand the pandemic and vaccination rollout entirely. A recent Australian study by Mahimbo et al. concurrently found in their study of 37 refugees that misinformation from social media was a common factor for leading their participants to be reluctant to receive the COVID-19 vaccination [9]. With ongoing reported literature on this theme in both Australia and across the globe [16-18], there should be further efforts to develop appropriate social media campaigns and information from the Department of Health and the respective Australian states. Additionally, recruitment and screening of interpreters or members of the community with either language qualifications that meet the national standards, such as a National Accreditation Authority for Translators and Interpreters (NAATI) qualification or additional language training provided by government and health authorities, should be prioritized to reduce health inequalities among CALD communities further.

Given the age range and demographic of the study, a unique perspective of this study includes members of the Australian community who recognize and could bridge the gap between the elderly and non-English speakers of their respective communities. Another aspect to consider is that CALD communities are diverse in their experiences, where refugee participants will have a different experience and perspective compared to participants from skilled migrant backgrounds or diaspora from different migrant families. Given the multicultural landscape, studies need to understand issues of CALD communities through a more diverse lens to reach as many different communities as possible rather than particularly one group alone, especially in a health emergency setting such as the COVID-19 pandemic.

Additionally, almost half the participants of this study either had previous experience or are currently working in the healthcare sector from a CALD background. The lack of health literacy among CALD communities and families was discussed among these participants across the two focus groups. From these discussions, along with the supporting literature from Liddell et al. regarding health literacy, it may be essential to assess the health literacy of CALD communities further [8], as the level of general health literacy may be different across the different nations migrants arrive from in comparison to Australia. Further assessment of creating appropriate materials, promotion of healthcare messaging, and resources offered to newly arrived migrants to Australia may be required to alleviate the gaps between health literacy and understanding of the Australian healthcare system.

Furthermore, assessing the experiences and understandings of the COVID-19 pandemic and vaccination rollout in healthcare workers of CALD communities across Australia may be a beneficial area to explore, given the reported lack of cultural awareness, ongoing translation barriers, and mistrust with information from the government and health authorities that CALD patients experience in this setting. Possible ways to address these issues include community-led initiatives in liaison with health authorities, including information sessions or public question-and-answer sessions with healthcare workers from CALD communities and cultural or religious leaders. These initiatives can help with the challenges in understanding and mistrust between health or government authorities from CALD communities. Additionally, having coordinators in healthcare settings for different CALD patients to guide patients to use services that can aid in their understanding of healthcare issues and make informed decisions about their health would alleviate many of the disparities. 

The interview method and sampling of participants have some possible limitations. Snowball sampling does have a risk of sample bias given the lack of random selection and, therefore, may not have been entirely representative of the population in question for the study. The study sample was more biased toward healthcare workers, where eight out of the 12 participants were better qualified and educated about COVID-19 and vaccinations, which may have impacted the development of other essential themes relatable to non-healthcare workers. Another limitation is that the study was conducted in English through English-speaking family members of people from communities struggling the most with language and translation barriers and are, therefore, only partially represented in this study. Furthermore, older members of the population and populations outside of Melbourne, Victoria, are not well represented in this study. Additionally, focus groups can be challenging, as participation among participants will be variable, and some voices may be underrepresented in the discussions, which may further add to the limitation of the study. Zoom sessions are more challenging than face-to-face focus group sessions due to technical challenges and a lack of facial or body expressions, which can add value to qualitative research. However, this study was well positioned to identify the translation barriers of health information during the pandemic as participants were often multilingual, and many worked within the healthcare sector. To address the study limitations, future research could consider larger sample sizes, with additional sampling methods such as purposive sampling, quantitative findings to complement the qualitative findings, further intersectional analysis, and language-specific data collection for further nuanced insights.

## Conclusions

In conclusion, CALD communities are continuously impacted negatively compared to the general population within health settings. This study explores the nuance of health implications among adults in CALD communities across Australia and within the healthcare system during the COVID-19 pandemic. Many experiences were similar across the three different states of the study regarding the negative experiences of healthcare access, community and family disagreements, and challenges with the government policies and vaccination rollout. However, Melbourne notably had more challenges across their migrant communities with both the length of time the state was in lockdown and the fluctuating government policies that intentionally targeted those of CALD backgrounds and lower socioeconomic status. It has been well documented that CALD communities were disadvantaged or neglected during the COVID-19 pandemic and vaccination rollout globally, particularly in the UK. However, this study highlights a similar phenomenon in the Australian setting, further consolidating the existence of health disparities among the varied migrant populations. Acknowledging these challenges and prioritizing the development of appropriate healthcare policies by the government and health authorities is imperative to prevent further exacerbation of adverse health outcomes among CALD populations, as they play an integral part in Australian society.
